# Derivation, Characterization, and Neural Differentiation of Integration-Free Induced Pluripotent Stem Cell Lines from Parkinson’s Disease Patients Carrying SNCA, LRRK2, PARK2, and GBA Mutations

**DOI:** 10.1371/journal.pone.0154890

**Published:** 2016-05-18

**Authors:** Olga Momcilovic, Renuka Sivapatham, Tal Ronnen Oron, Morten Meyer, Sean Mooney, Mahendra S. Rao, Xianmin Zeng

**Affiliations:** 1 Buck Institute for Research on Aging, Novato, CA, United States of America; 2 University of Southern Denmark, Odense, Denmark; 3 NxCell Science, Novato, CA, United States of America; 4 XCell Science, Novato, CA, United States of America; UCL Institute of Neurology, UNITED KINGDOM

## Abstract

We report generation of induced pluripotent stem cell (iPSC) lines from ten Parkinson’s disease (PD) patients carrying *SNCA*, *PARK2*, *LRRK2*, and *GBA* mutations, and one age-matched control. After validation of pluripotency, long-term genome stability, and integration-free reprogramming, eight of these lines (one of each *SNCA*, *LRRK2 and GBA*, four *PARK2* lines, and the control) were differentiated into neural stem cells (NSC) and subsequently to dopaminergic cultures. We did not observe significant differences in the timeline of neural induction and NSC derivation between the patient and control line, nor amongst the patient lines, although we report considerable variability in the efficiency of dopaminergic differentiation among patient lines. We performed whole genome expression analyses of the lines at each stage of differentiation (fibroblast, iPSC, NSC, and dopaminergic culture) in an attempt to identify alterations by large-scale evaluation. While gene expression profiling clearly distinguished cells at different stages of differentiation, no mutation-specific clustering or difference was observed, though consistent changes in patient lines were detected in genes associated mitochondrial biology. We further examined gene expression in a stress model (MPTP-induced dopaminergic neuronal death) using two clones from the *SNCA* triplication line, and detected changes in genes associated with mitophagy. Our data suggested that even a well-characterized line of a monogenic disease may not be sufficient to determine the cause or mechanism of the disease, and highlights the need to use more focused strategies for large-scale data analysis.

## Introduction

Parkinson's disease (PD) is a chronic, progressive and devastating neurodegenerative disorder, characterized by a profound loss of nigrostriatal dopaminergic neurons and accumulation of misfolded α-synuclein protein aggregates called Lewy bodies in remaining dopaminergic neurons and in other brain areas such as the cortex [1]. Clinical motor symptoms manifesting at early stages of the disease are often followed by non-motor symptoms such as autonomic dysfunction, mood alterations, and cognitive impairment in more advanced stages [2].

The etiology of PD includes a combination of genetic, environmental and, likely, epigenetic factors. While the majority of PD cases are idiopathic, about 15% of patients have a first degree relative affected with PD. Multiple genes, including *PARK2*, *LRRK2*, *GBA*, *SNCA*, *DJ-1*, *and PINK-1*, have been linked to familial forms of PD (for review see [3–6]). The most common monogenic PD-associated mutation is a G2019S substitution in *LRRK2* that causes the neurotoxic gain of function of LRRK2 protein kinase [7,8]. Many autosomal recessive mutations in *PARK2* have been detected and account for the most early onset PD cases [5]. Mutations in *GBA* that are the causative factor for Gaucher disease, a lysosomal storage disease, are also associated with Lewy body pathology and PD [9].

Of the familial PD genes, SNCA (α-synuclein) is of a particular interest since the SNCA protein is a major contributor to formation of Lewy bodies, a characteristic hallmark of PD at the cellular level [10]. SNCA is found in presynaptic vesicles and is implicated in neurotransmitter release, vesicle turnover, and channel localization [11]. Autosomal dominant *SNCA* triplication results in increased expression of α-synuclein and formation of neurotoxic α-synuclein aggregates, leading to earlier onset and faster disease progression [3,12]. α-synuclein protein aggregates are also detected in other neurodegenerative disorders collectively known as synucleinopathies [13], suggesting the aberrant clearance of aggregated proteins is a common neurotoxic pathway. It has been hypothesized that protein aggregation and defects in ubiquitin proteasome function lead to deficient aggregate removal and build-up of oxidative species generated by mitochondrial electron transport chain and by tyrosine hydrolysis in dopaminergic neurons [14]. Indeed, α-synuclein aggregates lead to overexpression of markers of oxidative stress and increased sensitivity to peroxide-induced oxidative stress, mitochondrial pathology, and subsequent cell death [14,15]. Additionally, *SNCA*-overexpressing skin fibroblasts exhibit decreased mitochondrial membrane potential, lowered ATP production, and reductions in mitochondrial complex I activity [16]. These findings suggest that α-synuclein is a modulator of oxidative damage and that the excess of SNCA associated with PD confers sensitivity to mitochondrial toxins. Not surprisingly, overexpression of human *SNCA* in transgenic mice instates mitochondrial dysfunction following treatment with the Parkinsonian neurotoxin MPTP (1-methyl-4-phenyl-1,2,3,6-tetrahydropyridine) [17].

One of the obstacles to studying PD is the inaccessibility of the affected brain tissues for studying molecular processes underlying the loss of dopaminergic neurons. Animal studies and overexpression of mutant proteins in cell lines are often inadequate, exemplifying the need for better disease models. To that end, induced pluripotent stem (iPSC) technology [18,19] makes it possible to generate iPSC lines from patients that can be differentiated into a cell type of interest, offering an unprecedented opportunity to study the cellular phenotypes that underlie disease [8,14,20–28].

In the present manuscript, we describe the derivation, characterization, and neural differentiation of eight integration-free iPSC lines derived from seven PD patients carrying various mutations, including *SNCA*, *LRRK2*, *PARK2*, and *GBA*, and an age-matched control. Whole-genome expression profiling of each line at different stages of differentiation showed no significant difference amongst the lines, highlighting the importance of developing isogenic controls. Nevertheless, focused examination of genes related to specific pathways, such as mitophagy, revealed alteration between patient and control lines. Given the importance of SNCA in PD etiology, we specifically examined gene expression in the SNCA line (two clones derived from the same patient) followed by MPTP treatment and found significant changes in genes associated to mitochondrial biology and cell death. We believe the lines and datasets we have generated will be a valuable resource for use in PD disease modeling and for the development of novel therapeutics.

## Materials and Methods

### Fibroblast and iPSC culture and differentiation

PD patient fibroblasts were obtained from the NINDS collection at the Coriell Institute for Medical Research (Camden, NJ; http://ccr.coriell.org). Fibroblasts were grown in Minimum Essential Medium Alpha, supplemented with 10–15% (line-specific) fetal bovine serum (FBS), and 1% antibiotic/antimycotic (all from Invitrogen, Carlsbad, CA; http://www.invitrogen.com) under conditions of 3% O_2_, 5% CO_2_ at 37°C in a humidified chamber, and passaged every 3–4 days using TrypLE^™^ (Invitrogen). iPSC maintenance, embryoid body formation and random differentiation, NSC derivation, and dopaminergic differentiation were carried out as previously described [29,30] For short tandem repeat (STR) and gene expression analyses, Pluripotent Stem Cell (PSC) were cultured on Matrigel^™^ human ES cell qualified Matrix (BD Bioscience, Bedford, MA; http://www.bdbiosciences.com) in mTeSR^™^1 medium (StemCell Technologies, Vancouver, BC, Canada; http://www.stemcell.com) supplemented with 1% antibiotic/antimycotic (Invitrogen), and passaged using Dispase (StemCell Technologies) according to manufacturer’s instructions. The cordblood iPSC derived L1 line, a subclone to XCL1, previously described [31].

### Derivation of integration-free iPSCs

Reprogramming using Sendai virus (SeV, CytoTune^™^ SeV kit, Invitrogen) was performed according to manufacturer’s recommendations. Briefly, 5x10^5^ fibroblasts were plated onto 35 mm dishes one day prior to SeV transduction. On day 1, fibroblasts were transduced with a mixture of 4 SeV carrying reprogramming factors (OCT4, SOX2, KLF4, and CMYC) and incubated for 24 hours. SeV-containing medium was replaced with fresh fibroblast growth medium on day 2, and medium was replenished every other day for a total of 6 days. On day 7, fibroblasts were transferred onto mouse embryonic fibroblast (MEF)-coated dishes at 5–5.5 x10^3^ cells/cm^2^ density (150x10^3^ cells per 60 mm dish), and a sample of 100–200 x10^3^ cells was set aside for RNA extraction (used as a positive control for the presence of SeV genome in iPSC clones). After two days (day 9), fibroblast medium was replaced with PSC medium consisting of KnockOut^™^ Dulbecco’s modified Eagle’s medium/Ham’s F12 supplemented with 20% KnockOut^™^ serum replacement, 1% nonessential amino acids, 1% GlutaMAX^™^, 1% antibiotic/antimycotic, 0.1 mM 2-mercaptoethanol (all from Invitrogen), supplemented with 10 ng/ml FGF2 and 0.25 mM sodium-butyrate (both from Stemgent, Cambrige, MA; https://www.stemgent.com/). Media was changed every other day until colonies appeared. TRA1-60 staining of live cells was performed to identify fully reprogrammed iPSC clones three to four weeks after SeV transduction. Single TRA1-60-positive colonies were manually dissected and transferred to fresh MEF-coated wells of a 12-well plate. The FGF2 concentration was gradually reduced to 4 ng/ml and clones were manually passaged and expanded into larger dishes.

### Short tandem repeat (STR) analysis

Genomic DNA was isolated from fibroblasts and corresponding iPSC lines using a Blood & Cell Culture DNA Mini kit (Qiagen, Valencia, CA; http://www.qiagen.com/) per manufacturer’s instructions. Samples were analyzed using a StemElite kit (Promega, Madison, WI; http://www.promega.com/) at the Fragment Analysis Facility at Johns Hopkins University (http://faf.grcf.jhmi.edu/). PCR products and appropriate positive and negative controls were electrophoresed on an ABI Prism^®^ 3730xl Genetic Analyzer using an Internal Lane Standard 600 (Promega). Data were analyzed using GeneMapper^®^ v 4.0 software (Applied Biosystems).

### Immunocytochemistry

Immunocytochemistry and staining procedures were performed as previously described [30,32]. Briefly, cells were fixed with 4% paraformaldehyde for 10 minutes, blocked in buffer containing 8% goat serum, 1% BSA, 0.1% Triton X-100 (all from Sigma, St. Louis, MO; http://www.sigmaaldrich.com) at room temperature for 1 h, followed by incubation with the primary antibody in blocking buffer at 4°C overnight. Primary antibodies were detected using species-specific fluorescently labeled secondary antibodies (Invitrogen). All secondary antibodies were tested for cross reactivity and non-specific immunoreactivity. The coverslips were mounted using Prolong-gold supplemented with DAPI (Invitrogen). The following primary antibodies were used: TRA1-60 (14-8863-82, eBioscience, 1:50) Nanog (14-5768-82, eBioscience, 1:50), OCT4 (Cell Signaling, 1:1200), SOX2 (MAB4343, Millipore, 1:500), AFP (A8452, Sigma, 1:1000), SMA (A2547, Sigma, 1:1000), SOX1 (BD Transduction Laboratories, 1:250), PAX6 (AB5790, Abcam, 1:500), Nestin (611658, BD Transduction laboratories, 1:500), Nestin (PRB-570C, Covance, 1:1000), β-III-tubulin (TUBB3; clone SDL.3D10, T8660, Sigma, 1:1000), TH (P40101, Pel-Freez, 1:500),FOXA2 (AB40874, Abcam, 1:1000), and LMX1A (AB31006, Abcam, 1:1000). The activity of alkaline phosphatase was tested using an alkaline phosphatase staining kit II (Stemgent) according to the manufacturer’s instructions. Quantification of immunoreactive cells in culture was performed by analyzing a minimum of 3000 cells in at least 10 randomly chosen fields derived from 3 or more independent experiments. The number of DAPI labeled nuclei on each image was used as a total cell number (100%).

### Gene expression analysis

Total RNA was isolated using the RNeasy Mini Plus kit according to the manufacturer’s instructions (Qiagen). For microarray analyses, RNA was hybridized to Illumina Human HT-12 BeadChip v4 (Illumina, Inc., San Diego, CA; http://www.illumina.com/) at the Microarray core facility at the Burnham Institute for Medical Research (La Jolla, CA; http://www.sanfordburnham.org). Data processing was performed using the Illumina GenomeStudio software. The background was subtracted and quantile method was used for normalization. The detection *p*-value for each transcript was a measurement of confidence that the transcript was expressed above the background (negative control probes). Dendrogram was constructed by global array clustering of genes across all tested samples by complete linkage method. All cell line correlations were a measure of Pearson's coefficient, implemented in *R* System.

For validation of the microarray results by quantitative PCR, one microgram of total RNA was used for the synthesis of complimentary DNA (cDNA) using iScript cDNA Synthesis kit (Bio-Rad, Hercules, CA; www.bio-rad.com/) according to the manufacturer’s recommendations. Quantitative PCR reactions were carried out on the CFX96^™^ Touch Bio-Rad instrument (Bio-Rad) using iTaq^™^ Universal SYBR^®^ Green supermix (Bio-Rad) following the manufacturer’s instructions. PCR reactions were conducted in duplicate or triplicate for each sample. Genomic DNA contamination and RNA quality were assayed using PrimePCR^™^ control assays (Bio-Rad). *TBP*, *GAPDH*, and *ACTB* were amplified as internal standards. Fold changes were calculated using the ΔΔCt method and normalized against endogenous *ACTB* (pluripotency and SeV genes) or *TBP* and *GAPDH* (NSC and DA gene expression). Primer sequences are listed in S1 Table.

### Pluritest

Pluritest is an online tool used to verify pluripotency. A stem cell matrix consisting of 450 genome-wide transcriptional profiles of diverse stem cells and differentiated cell types, as well as developing and adult human tissues from multiple laboratories, was generated using Ilumina microarrays. Among the 450 transcriptional profiles are 223 human ESC and 41 iPSC lines. Two classifiers were developed to obtain pluripotency and novelty scores. The pluripotency score reports to what extent the tested sample contains the pluripotency signature. The novelty score measures the technical and biological variation and reports the extent at which the measured signal in the test sample can be explained by the normal pluripotent stem cells [33]. The pluripotency score is a measurement of the similarity of the test sample to known PSC.

### Statistical analysis

Statistical significance was calculated by one-way analysis of variance (ANOVA) using Dunnett’s correction. *P<0.05, **P<0.01.

## Results

### Generation and characterization of integration-free PD patient-specific iPSC lines

PD patient fibroblasts carrying defined PD-associated mutations and age-matched healthy control fibroblasts were obtained from the National Institutes of Neurological Diseases and Stroke (NINDS) collection deposited at the Coriell Institute[34]. Line codes, PD-associated genotype, and epidemiological data are listed in Table 1. We will deposit the derived PD iPSC lines with Coriell.

**Table 1:** Integration-free iPSC lines generated from PD patient and control fibroblasts.

Gene	NINDS Catalog ID (Line code used in this study)	Mutation	Gender	Race	Age of PD	Age of sample	Family history
**SNCA**	ND27760 (A)	SNCA triplication	Female	Caucasian	50	55	Yes
**PARK2**	ND30171 (P)	PARK2: R42P	Male	Caucasian	42	54	No
		PARK2: EX3DEL					
	ND29543 (I)	PARK2: EX3-4DEL	Male	Hispanic	16	50	No
		PARK2: 1-BP DEL, 255A					
	ND29369 (B)	PARK2: R275W	Female	Hispanic	43	61	No
	ND31618 (S)	PARK2: R42P	Female	Caucasian	44	63	N/A
**LRRK2**	ND29802 (K)	LRRK2: G2019S	Male	Caucasian	40	52	No
	ND29492 (E)	LRRK2: G2019S	Male	Caucasian	58	72	Yes
	ND29542 (H)	LRRK2: G2019S	Male	Caucasian	34	57	No
**GBA**	ND31630 (T)	GBA: N370S	Male	Caucasian	63	69	No
	ND29756 (J)	GBA: N370S	Female	Caucasian	46	59	Yes
**Control**	ND34791 (Y)	Control	Female	Caucasian	n/a	60	Yes

**Fig 1:**
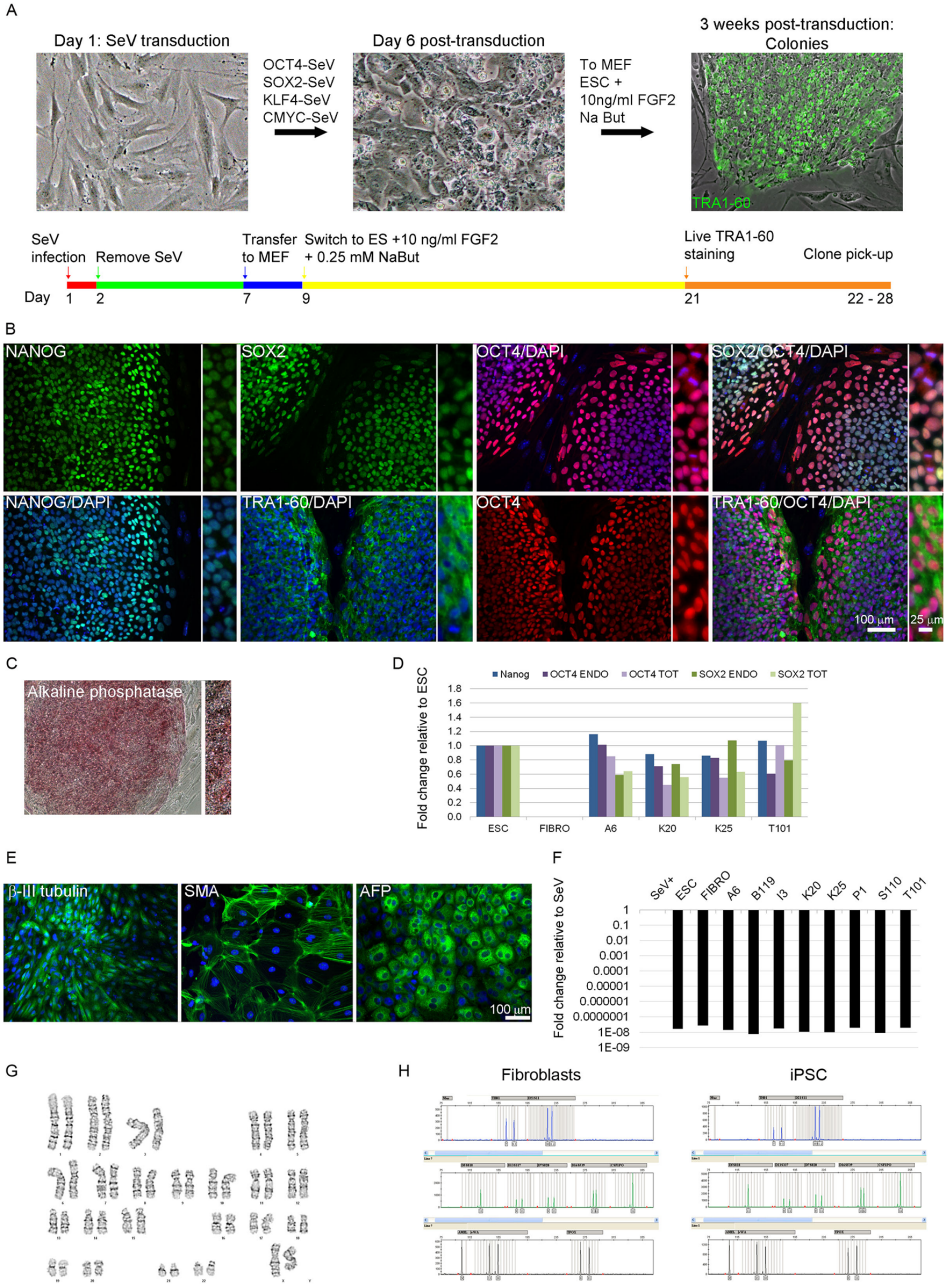
Characterization of patient-specific induced pluripotent stem cell (iPSC) lines. A: Schematic representation of the reprogramming process. B: Immunocytochemistry for pluripotency factors OCT4, NANOG, SOX2, and TRA1-60. C: Alkaline phosphatase reactivity. Inserts: Images taken at higher magnification. D: qPCR analysis of total and endogenous expression of pluripotency genes OCT4, SOX2, and NANOG. ACTB served as an endogenous reference, and data were normalized against ESC sample. E: Immunocytochemistry for markers of the three germ layers in embryoid bodies. Scale bar as marked. F: qPCR analysis of the expression of SeV-specific transcripts. *ACTB* served as an endogenous reference, and data were normalized against SeV sample (cells collected a week after transduction with SeV). Fold change is shown on logarithmic scale G: Karyotype analysis in *SNCA* triplication line A6. H: STR profiles of parent fibroblast and iPSC A6 line..

Fibroblasts were reprogrammed using a cocktail of 4 reprogramming factors, OCT4, SOX2, KLF4, and CMYC, carried by Sendai virus (SeV; Fig 1A). In order to ensure clonality of isolated iPSC lines, individual colonies were manually dissected and transferred to separate wells for further expansion and characterization. A representative iPSC characterization (A6 clone carrying *SNCA* triplication) and similar analysis of each iPSC line is reported in S1A–S1H Fig.

We first analyzed pluripotency by immunocytochemistry for OCT4, NANOG, SOX2, and TRA1-60, as well as alkaline phosphatase reactivity (Fig 1B and 1C). We then compared the endogenous and total expression of pluripotency factors *OCT4*, *SOX2*, and *NANOG* by quantitative PCR (Fig 1D). The embryonic stem cell (ESC) line H14 was used as a positive control, while parent fibroblasts served as a negative control. Characterization of I3, B119, S110 and P1 iPSC is reported elsewhere [31], and here we show qPCR data for A6, K20, K25, T101, and Y9. Differences in gene expression between ESC and iPSC, as well as amongst iPSC lines in total and endogenous expression of SOX2 and OCT4 were lower than 2-fold; in contrast, the fold difference between fibroblasts and pluripotent lines were on the 10^4^–10^5^ order of magnitude. These results indicate activation of the endogenous pluripotency network, consistent with the full reprogramming of patient fibroblasts.

We next examined the ability of the iPSC lines to differentiate into cells of the three germ layers via a standard embryoid body (EB) formation protocol [29,30]. The ability to differentiate *in vitro* was confirmed by the presence of ectoderm (β-III tubulin), mesoderm (smooth muscle actin, SMA), and endoderm (α-fetoprotein, AFP) in the EB (Fig 1E).

All lines were also validated for integration-free reprogramming at passage 15 via quantitative PCR using SeV-specific primers (Fig 1F). Non-transduced fibroblasts were used as a negative control, whereas cells collected one week after SeV transduction served as a positive control. In addition, genomic stability of each line was tested by karyotype analysis (Fig 1G) and only clones with normal diploid karyotype were used in subsequent experiments. We noted that all clones of one of the *GBA* mutant iPSC lines (T) carried a balanced translocation [46,XY,t(16;22)(p11.2;q11.2)], which was found in the patient fibroblasts as well (S1A–S1H Fig). Since no gross phenotypic abnormalities were present in the patient, we reasoned that this balanced translocation was silent, and included this iPSC line (T101) in our experiments.

Line identify was performed by short tandem repeat (STR) analysis in an independent facility (Fig 1H). DNA extracted from the parent fibroblasts and corresponding iPSC lines was sent to the Fragment Analysis Facility at Johns Hopkins University for the analysis. The STR profiles per investigated locus of parent fibroblasts and derived iPSC lines are listed in S2 Table.

Fully characterized, karyotypically normal iPSC clones (at least two different clones per patient fibroblast line) were banked. All clones were tested for mycoplasma contamination prior to banking. We intend to deposit all lines with the NIH and make them available to other investigators.

### Neural and dopaminergic differentiation of PD iPSC lines

**Fig 2:**
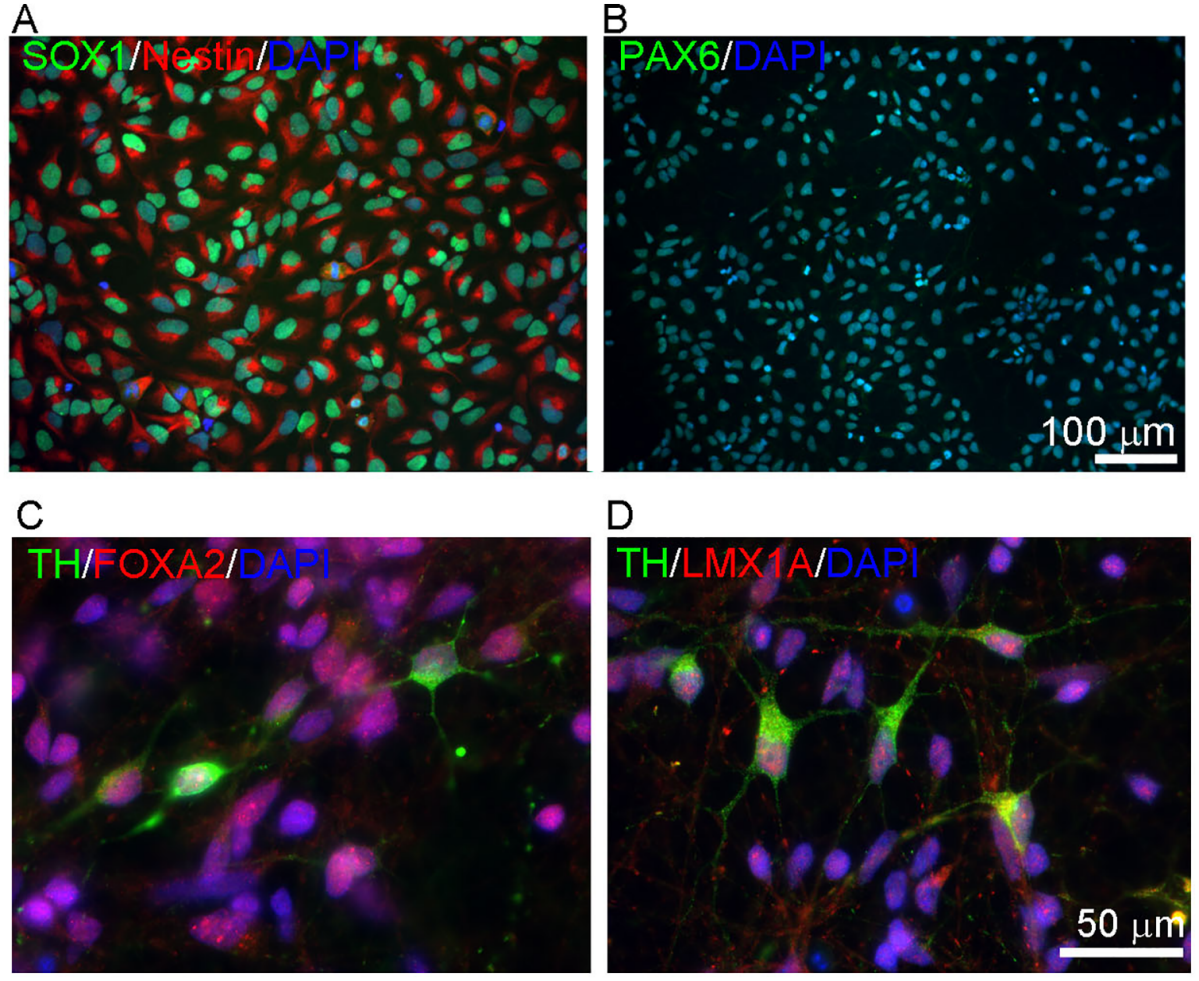
Neural and dopaminergic differentiation. A-B: Immunocytochemistry in *SNCA* triplication (A6) NSCs with antibodies against NSC markers SOX1, NESTIN, and PAX6. C-D: Immunocytochemistry for dopaminergic (TH and LMX1A), and midbrain (FOXA2) markers. Scale bar as marked.

We next determined whether these lines can be specifically differentiated into dopaminergic cultures using our stage-specific protocol [29], which has been used to generate more than 30 neural stem cell (NSC) lines from PSC [31,35–37]. All lines, except two of the three LRKK2 lines (E and H), could be differentiated into NSC and the timeline for NSC formation was similar between patient and control lines, and amongst the patient lines. NSC identify was confirmed by homogeneous expression of SOX1, Nestin, and PAX6 (Fig 2A and 2B). Characterization of each NSC line is reported in S1A–S1H Fig. Fig 2 shows representative images for the A6 line.

All NSC lines were able to differentiate into tyrosine hydroxylase (TH)-positive catecholaminergic/dopaminergic neurons using our standard protocol [29], albeit at varying efficiencies (Fig 2C). In order to confirm the midbrain origin of TH-positive cells, we performed co-immunostaining with antibodies against TH and FOXA2 (a floor plate marker; Fig 2C) as well as LMX1A (an early dopaminergic factor) (Fig 2D, from A6 line). Representative images of dopaminergic cultures differentiated from each line are reported in the S1A–S1H Fig.

### Whole genome expression profiling of PD patient-specific iPSC lines

A whole genome expression analysis was performed for all lines at fibroblast, iPSC, NSC, and dopaminergic culture stages, using Illumina Bead Array platform (Human HT-12 v4 Expression BeadChip). We have previously shown that this platform is suitable for reliable and robust detection of differential gene expression in a large number of samples [38]. For NSCs and dopaminergic cultures, at least two independent biological replicates per line were used for array analysis. The list of samples analyzed by microarrays is reported in S3 Table. Initial data processing was done in GenomeStudio software as previously described [38].

**Fig 3:**
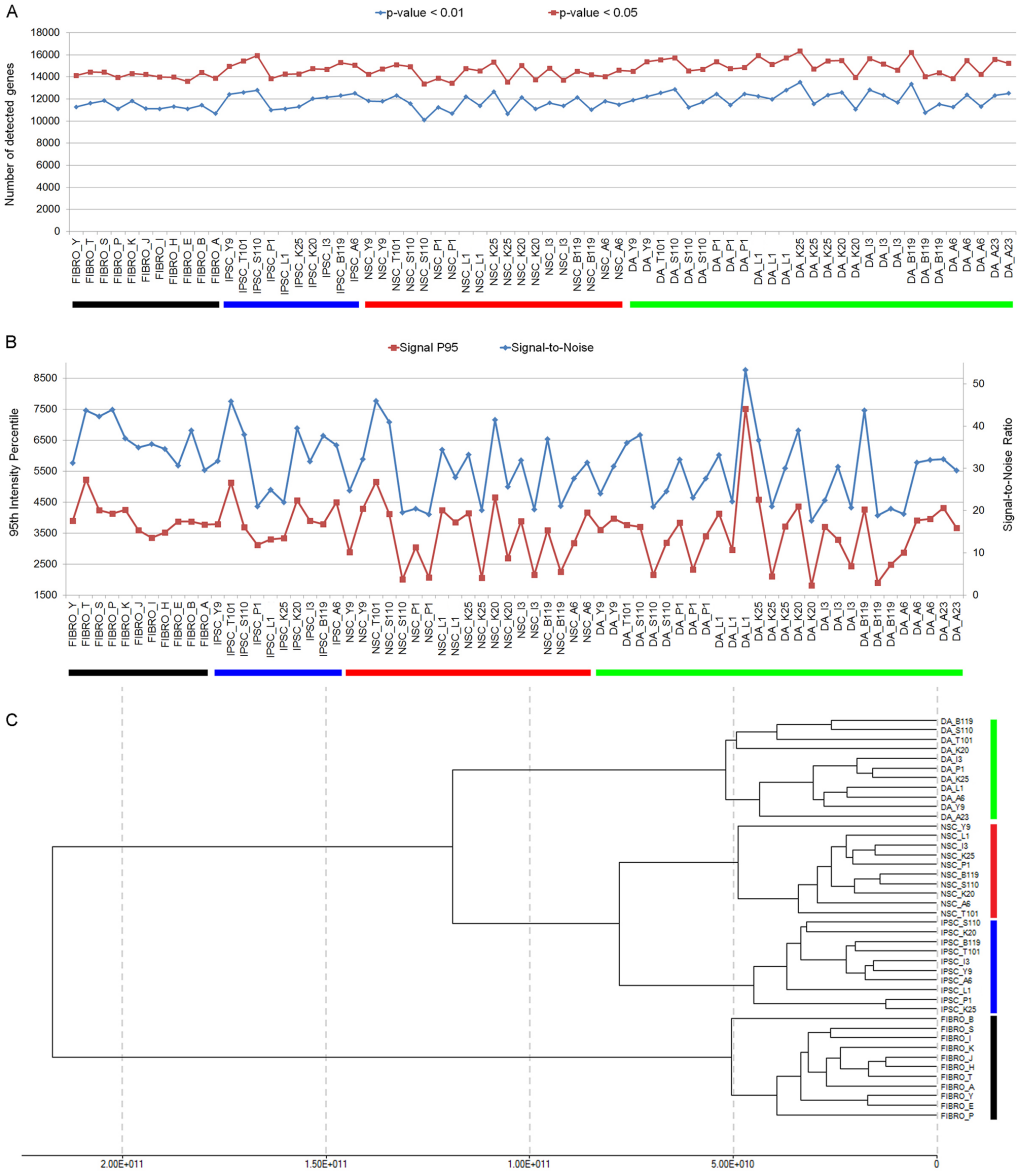
Microarray gene expression data quality control. A: the number of genes detected at p-value < 0.05 (red line) and p-value < 0.01 (blue line). Detection p-value is a measurement of confidence that a given transcript is expressed above the background level. B: Sample quality assessment by comparison of 95^th^ signal intensity values (red line) and signal-to-noise ratio (blue line) across samples. Signal-to-noise ratio is calculated as a ration of 95^th^ and 5^th^ percentile (p95/p05) in non-normalized data. C: Hierarchical clustering of samples after normalization and averaging of biological replicates.

Prior to further analyses, we assessed the quality of our data set. The average number of detected genes for all samples was highly similar: 11,798.4 ± 701.6 (detection *p*-value < 0.01; mean ± standard deviation; non-normalized data) and 14,661 ± 710.1 (detection *p*-value < 0.05; mean ± standard deviation; non-normalized data) (Fig 3A), and no wide discrepancies in hybridization signal intensity distributions were observed (not shown). In order to visualize the overall strength of measured signal across samples and identify presence of potential outliers, we plotted the signal to noise ratios and high-end intensity variation (95^th^ percentile of signal intensity, P95) (Fig 3B) in non-normalized data sets. Signal intensity was similar in all tested samples and no outliers were detected, suggesting matching quality across microarray samples.

After averaging biological replicates (two replicates per NSC line and 2–3 replicates for dopaminergic cultures), we calculated the pairwise correlation coefficients (*r*^2^) to determine the overall relatedness of samples (S4 Table). The correlation coefficients between samples at the same stage of development were ≥ 0.97, reflecting the stringent conditions under which the cell lines were derived and maintained. At the dopaminergic culture stage, however, *r*^*2*^ values were slightly lower (≥0.95), likely due to slight variations in culture conditions, variability within mixed cultures, and possibly differences in the genetic background. When comparing samples at different stages of the development, the level of relatedness was substantially decreased, as expected. In general, samples that were more closely related (such as, iPSC and NSC) showed higher *r*^*2*^ values than samples farther apart (iPSC and fibroblasts) (S4 Table). No mutation specific clustering was observed.

Next, we performed unsupervised one-way hierarchical clustering analysis to group averaged samples according to the degree of gene expression similarity (Fig 3C). The results displayed three distribution features: 1) iPSC and their differentiated derivatives clustered separately from the fibroblasts; 2) within iPSC and their neural progeny cluster, two subgroups were distinguished—stem cells and dopaminergic cultures; 3) within the stem cell subgroups, iPSC and NSC were clearly segregated in two categories. Thus, developmentally closer stages clustered closer together (iPSC and NSC) than those that were developmentally farther apart (fibroblasts and dopaminergic cultures) (Fig 3C), paralleling the conclusions from the coefficient of correlation results (S4 Table). We did not notice clustering, or higher coefficient of correlation values, for lines carrying mutations in the same gene at either developmental stage (for example, no clear clustering of *PARK2* mutant lines at fibroblast, iPSC, NSC, or dopaminergic culture stage). Most likely, the mutation-specific differences are so subtle that neither method for comparing overall relatedness of samples, such as coefficient of correlation and hierarchical clustering, can distinguish them beyond the level of cell type. We also performed clustering of biological replicates at the dopaminergic culture stage (2–3 replicates per line) separately from the other stages. When we initially normalized all samples (fibroblasts, iPSC, NSC, and dopaminergic cultures) together, we did not notice clustering of the biological replicates. This was not surprising as quantile normalization assumes equal signal distribution among samples, which was not the case when different developmental stages were compared. However, when we normalized dopaminergic culture replicates separately from the samples at other developmental stages, we detected clustering of samples carrying mutations in the same gene (data not shown). Therefore, in order to perform differential gene expression analysis, we normalized samples at different developmental stages—iPSC, NSC, and dopaminergic culture—independently of each other.

**Fig 4:**
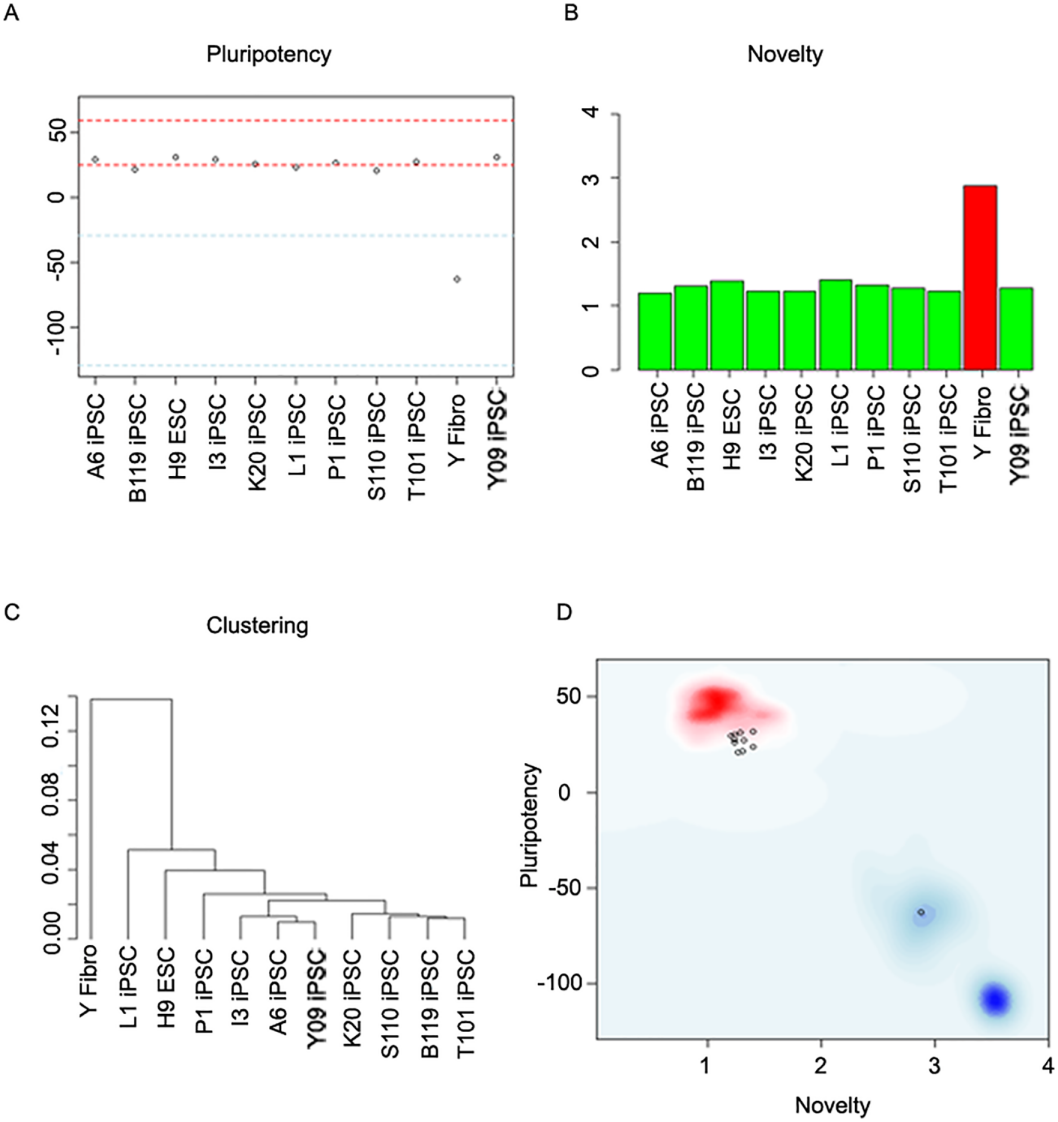
Pluripotency verification of PD patient iPSC. A: Pluripotency score for iPSC lines A6, B119, I3, K20, P1, S110, T101,L1 and Y09. ESC H9 line served as a positive control, whereas fibroblasts from the healthy subject (Y line) were used as a negative control. Pluripotency range is depicted in red, whereas non-pluripotent range is shown in blue. B: Novelty score for tested samples. Low novelty score (green bars) is characteristic for pluripotent cell lines, whereas high novelty sore (red) highlights sample that deviated from the pluripotent transcriptional signature. C: Hierarchical clustering of all samples. D: Combination of pluripotency and novelty scores illustrates that iPSC and ESC samples are grouped together (red background—high pluripotency and low novelty scores). Y fibroblast line had the opposite result (blue background—low pluripotency and high novelty scores).

We then examined the expression of known stage-specific genes in each line. The expression of pluripotency markers (S5 Table) was high in iPSC, similar to qPCR results (Fig 1D), whereas fibroblasts, NSCs and dopaminergic cultures lacked the expression of the same group of genes. To further confirm pluripotency, we used an online pluripotency method, Pluritest, as described in the Methods section. All iPSC lines and a positive control (H9 ESC) were located within or close to the 95% pluripotency range. As expected, the negative control (Y fibroblasts) was located in the non-pluripotent field (Fig 4A). Thus, all iPSC and ESC samples fell within the novelty score (green), whereas the fibroblast sample fell outside the novelty score (red) (Fig 4B). Hierarchical clustering clearly illustrated the similarity of transcriptional profiles between iPSC lines and the control ESC line (Fig 4C) [33]. Combination of novelty scores and pluripotency scores (Fig 4D) revealed that iPSC and ESC samples were grouped together (red cloud), suggesting an empirical distribution of pluripotent cells, whereas Y fibroblasts were located outside the pluripotent group (blue background; Fig 4D). Overall, Pluritest also demonstrated successful reprogramming of PD patient fibroblasts.

Multiple genes known to regulate neural induction including *SOX1*, *PAX6*, *SOX2*, *NES (Nestin)*, *CDH2*, *PROM1*, and *TMEM 88* (S5 Table), were highly expressed in NSC compared to iPSC or dopaminergic cultures. Genes associated with neurogenesis including *ASCL1*, *DCX*, *MAP2*, *NCAM 1*, *NCAM2*, *TUBB3*, *NEUROG2*, and *NEUROD1* were upregulated in dopaminergic cultures relative to iPSC and NSC (S5 Table). Furthermore, the expression of a subset of dopaminergic transcripts including *DRD1IP*, *EN1*, *EN2*, *FABP7*, *KCNJ6*, *NR4A2 (NURR1)*, and *TH* was high in dopaminergic cultures samples, but not at the other stages (S5 Table). Under the same cell culture conditions, the expression of non-ectodermal lineage genes was not detected (S5 Table).

**Fig 5:**
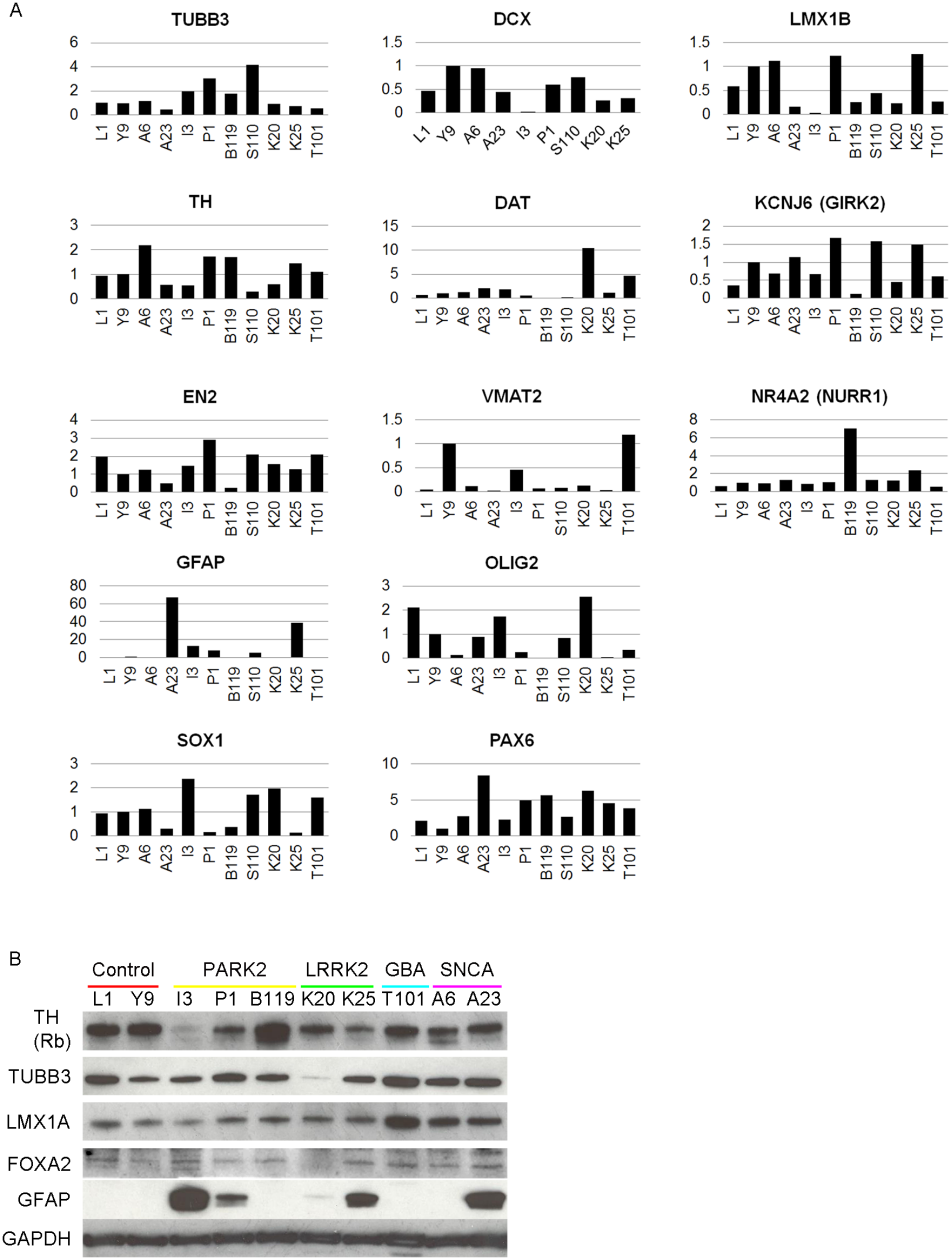
Dopaminergic differentiation of patient-specific neural stem cells (NSCs). A: qPCR validation of neuronal (*TUBB3*, *DCX*), NSC (*SOX1* and *PAX6*), glial (*GFAP* and *OLIG2*) and dopaminergic (*LMX1B*, *TH*, *DAT*, *GIRK2*, *EN2*, *VMAT2*, *NURR1*) gene expression in dopaminergic cultures. *TBP* and *GAPDH* were used as endogenous reference, and data were normalized against control line (Y9). B: Western blot analysis of TH, TUBB3, LMX1A, FOXA2 and GFAP in PD-patient specific dopaminergic cultures. GAPDH was used as a loading control. A CD34+ blood cord derived iPSC line L1 [31], was used as a control.

Expression of the several neuronal/dopaminergic (TUBB3, DCX, LMX1B, TH, DAT, GIRK2, VMAT, EN2, and NURR1), NSC (SOX1 and PAX6), and glial (GFAP and OLIG2) markers following dopaminergic differentiation was also validated by qPCR (Fig 5A). The level of TH, TUBB3, LMX1A, FOXA2, and GFAP protein expression was examined by western blot (Fig 5B). Our WB data showed less TH in I3 is consistent with previously published data. Collectively, qPCR and protein expression data show that all NSC lines could differentiate into dopaminergic cultures, as well as glial cells, although at varying efficiencies. The iPSC line L1, a subclone of XCL1 as previously described[31], was used as another control in Fig 5.

### Altered gene expression in dopaminergic cultures in patient lines and effect of MPTP

Although all NSCs derived from the PD patient iPSC lines differentiated into dopaminergic cultures and the overall gene expression profiles were similar, we reasoned that the expression of certain pathways might be altered in specific PD patient-derived dopaminergic cultures and warranted closer examination of the microarray data. We focused our analysis on one selected mutation, *SNCA* triplication, due to its complete penetrance and significant role in PD. SNCA triplication lines (A6 and A23) could be successfully differentiated to dopaminergic cultures, and we confirmed co-expression of TH and alpha-synuclein in A6 and control (Y9) dopaminergic cultures (S2A Fig). Quantitative PCR showed elevated SNCA expression in A6, but not A23 dopaminergic culture relative to control lines (S2B Fig). We also validated increased expression of alpha-synuclein in SNCA triplication relative to control lines at the protein level by Western blot (S2C Fig).

To identify genes that may be altered (3-fold or more) in the context of *SNCA* mutation, we compared the gene expression of dopaminergic cultures from the two *SNCA* triplication clones (A6 and A23) versus the control Y9 line. 700 genes were found to be expressed 3-fold or higher in the SNCA line, whereas 400 genes were expressed 3-fold or lower in both *SNCA* triplication clones. The top 50 upregulated and downregulated genes are presented in Table 2. As a further filter, we cross-referenced this list with genes similarly dysregulated in other familial PD lines. A list of genes that showed 3-fold upregulation, including *NNAT*, *CHCHD2*, *PTGR1*, and *NLRP2*, or downregulation, such as *IFITM1*, *IL13RA2*, *BGN*, and *NKX2-2* in PD patient-derived lines relative to healthy control is shown in Table 3. Given the link between mitochondrial biology and PD, we were particularly interested in examining the expression of the over 600 genes related to mitophagy, cell stress, and cell death in our microarray data. As seen in Table 4, 14 genes including *PMAIP1*, *ISLR2*, and *BID* were up-regulated over 3-fold in both SNCA clones, while 18 genes including *MMP*, *CASP1*, and *HRK* were down-regulated 3-fold or more in the two *SNCA* triplication clones.

**Table 2:** Gene up or down regulated in *SNCA* triplication dopaminergic cultures relative to healthy control.

SYMBOL	A6	A23
**Genes upregulated 3 fold in *SNCA* triplication dopaminergic cultures relative to healthy control**.		
**NNAT**	972.38	902.44
**CHCHD2**	597.65	464.43
**LOC728324**	65.99	23.06
**CHCHD9**	63.61	27.65
**LOC645317**	38	26.7
**CYP26B1**	29.03	1.24
**LOC646630**	23.06	10.44
**PMP2**	21.76	16.48
**MGMT**	20.16	33.3
**S100A6**	19.03	50.32
**ZNF252**	18.52	14.16
**PHACTR3**	18.01	9.2
**TSPAN1**	16.5	2.02
**Hs.552826**	14.91	1
**ASTE1**	13.9	6.17
**TAF9L**	13.49	6.24
**OTP**	13.43	3.04
**NLRP2**	13.42	1.08
**SP8**	13.32	17.58
**CLEC11A**	13.27	18.06
**LIN28B**	11.98	4.77
**ZNF439**	11.8	1
**TUBB4**	11.7	12.64
**Hs.551307**	11.27	6.34
**CSRP1**	10.83	1.67
**SYT3**	9.83	16.03
**KHDRBS2**	9.71	1
**PCDHB5**	9.57	0.37
**ART3**	9.08	1.1
**FAM71E1**	8.84	13.5
**HOXB8**	1	339.79
**HOXB7**	1	168.8
**HOXA9**	1	129.7
**MSX1**	3.41	120.64
**NKX6-2**	6.48	90.63
**HOXB5**	0.29	68.93
**C8orf55**	1	61.44
**LHX3**	1	60.27
**HEY2**	4.64	59.37
**HOXC9**	1	49.98
**HOXC4**	1	42.9
**HOXA5**	0.11	41.84
**HOXD11**	1	40.32
**Hs.19193**	1	38.51
**HOXC8**	1	37.89
**HOXD10**	1	34.4
**PAX6**	4.07	34.16
**H19**	3.5	32.79
**NXPH2**	2.36	31.96
**Genes downregulated 3 fold in *SNCA* triplication dopaminergic culture relative to healthy control**.		
**IFITM1**	0.04	0.02
**FLJ30058**	0.05	0.02
**C21orf63**	0.06	2.05
**SLC15A4**	0.06	0.12
**MAP1LC3C**	0.07	0.05
**GAL**	0.08	0.08
**PERP**	0.08	0.11
**EDNRA**	0.09	0.05
**CLDN5**	0.1	0.2
**ESPN**	0.1	0.22
**C1QTNF1**	0.1	0.08
**CALCRL**	0.1	0.47
**TMOD1**	0.1	2.35
**NFIX**	0.11	0.58
**EFHC2**	0.11	0.54
**TMEM132D**	0.11	0.67
**HOXA5**	0.11	41.84
**BMP5**	0.11	0.07
**EFHD1**	0.11	0.49
**TCERG1L**	0.11	0.11
**GAPDHL6**	0.12	0.12
**KCNK12**	0.12	1.11
**OLFML2B**	0.12	0.03
**PDGFRB**	0.12	0.13
**PHOX2B**	0.12	0.12
**COL3A1**	0.12	0.05
**LYN**	0.13	0.11
**A2M**	0.13	0.2
**SNTB1**	0.13	0.08
**TFPI2**	0.13	0.13
**ZIC2**	0.24	0.02
**POU4F2**	1.08	0.03
**DOCK10**	0.42	0.04
**CDH10**	1.44	0.04
**CYP26A1**	0.43	0.05
**CBLN2**	0.53	0.05
**LHX2**	0.86	0.06
**HAS3**	0.78	0.06
**SMOC1**	0.79	0.06
**CTSC**	0.52	0.07
**C1orf59**	2.85	0.07
**COL1A2**	0.25	0.07
**VGLL3**	0.3	0.07
**TAC1**	1.53	0.07
**SFTA1P**	1.07	0.08
**SLC10A2**	0.15	0.08
**LRRC4C**	0.79	0.08
**NGB**	0.36	0.08
**FBLN5**	0.26	0.08

**Table 3:** Genes up or down regulated in all PD-patient specific dopaminergic cultures relative to control.

**Genes upregulated 3-fold in all PD-patient specific dopaminergic cultures relative to control**.									
**SYMBOL**	P1 DA	I3 DA	S110 DA	B119 DA	A6 DA	A23 DA	K20 DA	K25DA	T101 DA
**NNAT**	609.99	322.63	696.56	0.64	882.19	870.90	933.17	500.50	704.26
**CHCHD2**	439.76	390.55	1.40	292.05	773.18	501.20	473.31	14.79	5.91
**PTGR1**	17.99	13.90	1.85	5.48	7.22	5.03	27.50	12.63	12.30
**NLRP2**	14.50	23.41	14.16	0.11	18.41	3.19	11.58	16.29	9.53
**ID1**	11.41	4.85	0.86	8.69	3.01	4.79	12.59	19.61	20.04
**LOC645550**	7.02	2.50	3.95	5.26	6.37	9.66	4.80	9.63	3.70
**SLC30A3**	6.85	4.51	2.35	4.02	3.60	8.77	0.70	4.56	3.48
**LOC646858**	6.50	2.31	2.12	2.30	3.27	3.36	8.43	6.85	6.33
**HS.563340**	6.07	2.71	2.35	1.86	2.18	3.37	7.66	6.97	5.32
**LOC100133099**	5.55	3.58	3.23	3.96	3.54	4.27	3.83	7.13	6.68
**ZNF491**	5.49	2.17	5.52	4.97	7.14	2.94	4.81	5.18	5.93
**SCML1**	5.40	3.50	2.89	3.59	3.07	4.37	3.47	3.72	6.52
**FLJ10916**	5.24	4.08	2.15	4.59	3.00	3.46	3.42	2.76	2.16
**LOC100132648**	5.11	2.72	3.07	2.48	3.87	3.63	3.22	5.92	3.43
**HIST1H2BD**	5.03	2.34	2.35	3.10	3.51	1.53	4.39	2.44	2.32
**ZSCAN5A**	4.97	7.63	3.48	4.22	4.11	4.93	10.52	4.99	6.79
**LOC100132048**	4.90	2.89	3.54	2.96	3.70	3.05	3.00	5.35	3.91
**ACOT2**	4.71	5.15	3.83	5.95	4.44	8.32	11.06	6.88	9.06
**ME3**	4.46	3.66	3.49	8.24	0.61	3.96	4.92	6.95	7.89
**ACOT1**	4.42	4.32	3.53	5.31	3.49	7.49	10.45	6.84	7.31
**LOC645863**	4.41	1.45	2.54	5.56	4.31	5.72	3.20	6.91	3.91
**IGF2BP1**	4.29	1.84	2.48	3.28	2.86	2.57	3.96	4.60	4.56
**HMBOX1**	4.15	3.05	3.92	3.48	3.20	4.21	4.70	3.69	6.79
**LOC645743**	4.13	1.86	2.28	2.21	2.84	2.92	3.01	5.01	5.64
**TUBB4**	4.05	2.51	3.98	6.24	6.02	8.49	1.52	4.48	10.18
**CSRP1**	4.04	11.61	11.73	17.17	13.65	4.90	18.79	29.61	23.19
**LOC729339**	3.92	2.29	1.74	2.02	2.72	3.71	2.20	4.50	2.60
**ZNF252**	3.76	4.18	5.33	5.11	5.44	5.68	2.26	3.31	7.17
**LOC642570**	3.75	3.01	3.18	2.14	2.92	2.39	2.90	3.16	2.78
**RWDD2B**	3.70	4.65	1.59	3.89	4.14	4.66	3.84	5.57	5.49
**ASTE1**	3.58	3.69	4.63	5.67	12.49	5.69	14.95	6.99	0.03
**ZNF630**	3.49	6.71	2.87	3.47	2.93	1.17	2.92	2.96	2.98
**B3GALNT1**	3.28	5.31	6.37	9.82	4.49	5.97	5.34	6.33	8.17
**Hs.317248**	3.27	1.87	4.75	2.77	3.70	2.26	2.83	3.76	2.99
**LOC100131835**	2.91	2.63	3.63	3.62	1.37	2.55	3.16	3.86	5.13
**ZNF333**	2.72	1.48	2.90	2.42	2.32	2.68	2.92	2.52	3.37
**ZNF333**	2.62	2.37	2.11	2.62	2.13	1.41	2.77	2.56	2.61
**HS.545993**	2.44	3.26	2.83	2.53	2.47	1.16	2.29	2.41	3.49
**LOC731954**	2.39	5.26	1.31	2.16	2.13	4.01	4.82	3.11	2.29
**S100A6**	2.29	3.77	1.90	4.26	3.44	6.89	10.59	5.74	2.32
**TAF9L**	2.15	2.55	2.31	1.69	2.65	2.16	2.65	2.70	3.84
**LOC100129117**	1.99	2.45	2.46	2.07	2.17	2.06	2.74	2.17	2.12
**Genes down regulated 3-fold in all PD-patient specific dopaminergic cultures relative to control**.									
**SYMBOL**	P1 DA	I3 DA	S110 DA	B119 DA	A6 DA	A23DA	K20 DA	K25 DA	T101 DA
**IFITM1**	0.01	0.05	0.02	0.01	0.07	0.04	0.85	0.01	0.01
**IL13RA2**	0.09	0.13	0.59	0.13	0.25	0.18	0.23	0.05	0.18
**NKX2-2**	0.00	0.02	0.00	0.00	0.05	0.01	0.01	0.06	1.03
**BGN**	0.08	2.48	0.01	0.04	0.05	0.16	0.16	0.08	0.00
**OLIG1**	0.00	0.15	0.03	0.00	0.01	0.04	0.07	0.01	0.11
**FLJ30058**	0.00	0.02	0.19	0.12	0.04	0.08	0.00	0.00	0.01
**OLIG2**	0.01	0.24	0.07	0.01	0.04	0.05	0.42	0.03	0.13
**S100A4**	0.04	0.05	0.01	0.01	0.05	0.07	0.08	0.07	0.03
**FEV**	0.01	0.08	0.02	0.00	0.04	0.02	0.02	0.05	0.78
**VGLL3**	0.26	0.08	0.28	0.00	0.31	0.01	2.27	0.00	0.11
**CACNG3**	0.04	0.09	0.04	0.04	0.46	0.26	0.20	0.02	0.07
**SLC7A10**	0.02	0.06	0.10	0.14	0.06	0.19	0.00	0.04	0.01
**TCERG1L**	0.00	0.00	0.00	0.00	0.00	0.00	0.00	0.04	0.37
**WFDC1**	0.00	0.04	0.00	0.02	0.00	0.01	0.01	0.01	0.14
**PHOX2B**	0.03	0.10	0.22	0.07	0.07	0.04	0.43	0.17	0.22
**LOC644366**	0.29	0.00	0.86	0.25	0.00	0.00	0.02	0.00	0.00
**LOC642639**	0.11	0.25	0.14	0.06	0.10	0.15	0.30	0.11	0.21
**PERP**	0.22	0.17	0.53	0.17	0.17	0.17	0.24	0.12	0.22
**BMP5**	0.13	0.14	0.05	0.10	0.20	0.07	0.63	0.07	0.00
**ZNF154**	0.07	0.24	0.16	0.04	0.21	0.11	0.44	0.12	0.02
**SLC10A2**	0.05	0.03	0.54	0.07	0.18	0.06	0.07	0.09	0.01
**FOXA1**	0.01	5.02	0.20	0.01	0.15	0.01	0.27	0.01	0.19
**C1QTNF1**	0.08	0.09	0.03	0.08	0.10	0.13	0.12	0.09	0.15
**KRT81**	0.01	0.04	0.03	0.01	0.35	0.05	0.08	0.10	0.10
**CRYBA2**	0.06	0.20	0.04	0.10	0.01	0.08	0.11	0.08	0.81
**SPATA17**	0.27	1.50	0.19	0.06	0.15	0.34	0.86	0.18	0.18
**CPA4**	0.08	0.18	0.24	0.09	0.38	0.26	0.24	0.15	0.01
**TPH2**	0.01	0.15	0.07	0.01	0.22	0.08	0.03	0.14	0.59
**PPY**	0.03	0.31	0.01	0.29	0.15	0.99	0.24	0.11	0.11
**APLNR**	0.01	0.37	0.17	0.01	0.03	0.10	0.15	0.01	0.01
**MYL4**	0.10	0.09	0.30	0.10	0.17	0.14	0.25	0.23	0.03
**APCDD1L**	0.01	0.04	0.05	0.08	0.09	0.01	0.08	0.02	0.01
**OR2L13**	0.02	0.03	0.02	0.16	0.22	0.19	0.06	0.28	2.11
**CD34**	0.05	0.27	0.16	0.12	0.55	0.18	0.26	0.08	0.12
**BARHL1**	0.02	0.02	0.08	0.02	0.15	0.02	69.77	0.02	0.14
**ITM2A**	0.19	1.21	0.16	0.07	0.05	0.22	0.44	0.26	0.18
**IL28RA**	0.02	0.23	0.14	0.27	0.02	0.45	0.02	0.02	0.16
**LOC728800**	0.02	0.02	0.13	0.02	0.05	0.02	1.82	0.02	0.02
**LOC643599**	0.02	0.02	0.24	0.02	0.02	0.02	0.02	0.94	0.02
**ATP2C2**	0.03	0.02	0.18	0.16	0.24	0.30	0.68	0.25	0.29
**C8G**	0.18	0.02	0.31	0.02	0.12	0.24	0.02	0.09	0.13
**LOC131909**	0.26	0.26	0.03	0.07	0.06	0.27	0.24	0.08	0.32

**Table 4:** Mitogenic genes up or down regulated 3 fold in *SNCA* triplication dopaminergic cultures relative to control.

SYMBOL	A6	A23
**Mitogenic genes upregulated 3 fold in *SNCA* triplication dopaminergic cultures relative to control**.		
**PMAIP1**	4.36	9.12
**ISLR2**	4.24	8.76
**BID**	3.63	3.09
**UCP2**	3.37	5.52
**NGFR**	1.32	10.44
**CDKN1A**	2.18	5.87
**NR4A2**	1.8	5.13
**RNU4ATAC**	0.78	4.86
**LOC100130276**	1.78	4.14
**RARA**	1.27	3.44
**SNCG**	1.59	3.37
**PSMA7**	1.83	3.37
**HIP1R**	1.81	3.18
**DPF1**	2.07	3.17
**Mitogenic genes downregulated 3 fold in *SNCA* triplication dopaminergic cultures relative to control**.		
**MMP2**	0.09	0.58
**CASP1**	0.09	-0.28
**MMP26**	0.2	0.33
**HRK**	0.24	1.58
**ATP1A2**	0.28	0.03
**ATG16L2**	0.29	0.64
**ATP6V0D2**	0.3	0.69
**LAMP2**	0.3	0.38
**ATP10B**	1	0.06
**ATP7A**	1.37	0.12
**MMPL1**	0.39	0.17
**LOC100133719**	0.51	0.2
**ALOX5AP**	0.52	0.23
**CASP7**	0.82	0.23
**CARD11**	0.38	0.23
**ATF3**	0.38	0.26
**ATF7IP**	0.67	0.26
**ATP2B4**	0.54	0.27

Since PD patient iPSC lines have been reported to be more susceptible to oxidative stress [26], we also analyzed gene expression changes in dopaminergic cultures carrying *SNCA* triplication in response to MPP-induced stress. After challenging dopaminergic cultures generated from control and *SNCA* triplication lines with MPP^+^ for 24 hours, as previously described [39], we performed whole genome expression analysis. The top 30 genes up-regulated by 3-fold out of 585 and 30 genes down-regulated out of over 1000 genes are shown in Table 5. In general, SNCA clones responded to MPP^+^ similarly to the control line. A down regulation in dopamine transporters and an increase in mitochondria associated cell death genes, Harakiri in particular [40–42], were observed in both *SNCA* triplication and control lines after MPP^+^ treatment (Table 5). Changes in the expression of other familial PD genes were modest. In particular, *LRRK2* expression was undetectable as has been previously reported [26]. These results indicated that there was no dramatic difference at the transcript level between SNCA and control lines after exposure to MPP^+^. We did note that *HSPA1B* and *HSPA1A* were significantly upregulated in one of the clones (A23) which had lower *SNCA* levels than the other clone (A6; Table 5).

**Table 5:** Genes up or down regulated 3 fold in control and *SNCA* triplication dopaminergic cultures after MPP treatment.

SYMBOL	Y09	A6	A23
**Genes upregulated 3 fold in control and *SNCA* triplication dopaminergic cultures after MPP treatment**.			
**GDF15**	194.5	265.8	234.95
**ADM**	2.27	4.83	62.44
**INHBE**	6.2	16.62	24.35
**CLDN1**	11.34	8.85	22.96
**STC2**	21.42	22.85	22.09
**HSPA6**	14.37	4.23	21.48
**Hs.538535**	2.97	6.18	20.13
**P8**	14.12	8.23	19.45
**HSPA1B**	2.24	1.38	19.31
**HSPA1A**	2.71	1.88	18.02
**HRK**	4.86	7.37	3.46
**ULBP1**	20.65	15.78	17.37
**NUPR1**	14.1	9.42	17.06
**GEM**	12.49	8.62	15.24
**DDIT3**	17.18	20.79	14.38
**NAMPT**	7.89	6.01	14.2
**EMP1**	4.97	10	14.15
**ITGA5**	2.85	5.68	13.78
**LOC648605**	6.64	6.38	13.78
**MTHFD2**	6.95	10.79	13.72
**SLC7A3**	2.86	9.17	13.45
**AGPAT9**	5.64	3.4	13.38
**RBM47**	13.88	5.51	13.15
**SLC1A5**	8.43	5.55	12.86
**SNORD25**	5.96	7.7	12.72
**SNORD3D**	4.9	2.78	12.62
**RORB**	6.02	2.55	12.13
**LAMP3**	4.46	8.12	12.12
**GADD45B**	1.64	1.23	11.82
**Genes downregulated 3 fold in control and *SNCA* triplication dopaminergic cultures after MPP treatment**.			
**Hs.192506**	0.23	0.07	0.01
**WDR81**	0.54	0.07	0.01
**ELAC1**	0.18	0.01	0.01
**ZNF287**	0.11	0.09	0.01
**WDR5B**	0.4	0.15	0.01
**AP4E1**	0.47	0.25	0.01
**UFSP1**	0.21	0.07	0.01
**C8orf30B**	0.28	0.21	0.01
**MAGOHB**	0.2	0.1	0.01
**ACACB**	0.28	0.11	0.01
**ERP27**	0.03	0.07	0.01
**LOC442075**	0.25	0.03	0.01
**ORAI3**	0.49	0.03	0.01
**HES2**	0.72	0.15	0.01
**GPR68**	0.29	0.74	0.01
**FLJ20464**	0.03	0.02	0.01
**RRP1**	0.84	0.27	0.01
**C11orf88**	0.03	0.33	0.01
**PSKH1**	0.02	0.03	0.01
**HIST1H1B**	0.03	0.46	0.01
**CLIC5**	0.03	0.03	0.01
**RAB36**	0.39	0.06	0.01
**PLEKHO2**	0.27	0.21	0.01
**Hs.26651**	0.17	0.09	0.01
**HSD3B7**	0.61	0.21	0.01
**Hs.545045**	0.02	0.05	0.01
**SLC18A3**	0.03	0.03	0.01
**LOC643466**	0.59	1.05	0.01
**CRSP6**	0.08	0.23	0.02

Overall, our data showed that clones carrying *SNCA* triplication could survive and be differentiated into dopaminergic cultures. Even though two *SNCA* triplication clones originated from the same parent fibroblast line (thus sharing the same genetic background), and both passed all the relevant QC tests, significant variations in dopaminergic differentiation (Fig 5), the level of *SNCA* expression (S6 Table) in their response to stress were detected (Table 5) Changes in gene expression observed in *SNCA* triplication clones were similar to those observed in control line following MPP^+^ treatment. This suggests that the MPTP assay is a useful initial screen and that drugs identified by this screen will likely work in patient lines carrying PD-associated mutations.

## Discussion

The etiologic of idiopathic PD remains unclear, but likely results from a complex interaction between plural genetic susceptibilities and environmental factors including pesticides, herbicides, and industrial chemicals. Although progress has been made, the fundamental understanding of the pathogenesis of PD required for the development of prophylactic measures and effective new treatments is still lacking. Even though the majority of PD cases are idiopathic, the effects of mutations associated with familial PD such as *SNCA*, *LRRK2*, *PARK2*, *PINK1*, and *GB*A are the focus of intense research [3–6]. There are several obstacles to studying PD despite the availability of mouse models and *in vitro* culture assays. First, there are no reliable biomarkers of PD that can be used for identification of patients at risk prior to the onset of the disease. Second, the causes of the death of dopaminergic neurons at the cellular level are largely unknown and potential therapeutic targets still need to be discovered. Third, a relevant disease model for studying the underlying pathological processes is still missing since affected neurons cannot be obtained from the PD patients (except for post-mortem tissue of limited value), and animal studies are often an inadequate representation of what occurs in human patients.

We and others have suggested that iPSC technology may provide the missing link [21,22,26–28,43,44]. Reprogramming of somatic cells into a pluripotent state and subsequent differentiation of iPSC into cell types of interest enables generation of live dopaminergic cultures with the genetic background of PD patients. Since iPSC can be expanded and differentiated *in vitro*, it is possible to generate large quantities of neurons for mechanistic disease modeling and for drug screening from both idiopathic PD cases and PD patients carrying known PD-associated mutations. Indeed, a number of iPSC from patients with idiopathic and familial forms of PD have been previously derived [21,22,26–28,43,44]. Despite these opportunities, the results from such studies have been variable and difficult to extend to standard screening models due to variability, including inconsistent functional properties of the resulting cells.

In the present study, we generated iPSC lines from ten PD patients carrying various mutations (1 *SNCA*, 4 *PARK2*, 3 *LRRK2*, and 2 *GBA*) and one age-matched healthy control subject. To reduce variability, we used an integration-free reprograming technique, passaged the cells at the iPSC stage for at least 15 passages, established that the expression of imprinted genes was normal (data not shown), and that the cells retained a normal karyotype. In addition, we confirmed all lines were competent to differentiate into ectoderm, endoderm, and mesoderm and that all lines fell in the same profile as normal ESC/iPSC lines in the Pluritest. To further reduce potential protocol-related variability over the time period required for the generation of dopaminergic cultures, we used an intermediate NSC step where we could confirm the quality of cells including their purity and homogeneity. This strategy had been of great use in our analysis of other CNS disorders including Niemann-Pick disease [36,45,46].

All lines described in this manuscript, with the exception of two *LRRK2* lines carrying the G2019S mutation, formed rosettes and NSC stocks could be established. The growth kinetics and gene expression profiling of NSC were similar for all lines. The fact that all NSC lines, could be differentiated into dopaminergic neurons in culture, suggests that the underlying biological defect did not affect early differentiation processes, even though the message for several PD related proteins were detected at the NSC stage. This illustrates both the importance of examining gene profiles at the appropriate developmental stage and the difficulties in using an iPSC-based system for a chronic disease.

To further reduce variability, we generated more than one clone from the same individual [22]. As an example, we derived two clones, A6 and A23, from the same *SNCA* triplication patient. We found no significant differences in the basal levels of expression of mitochondrial genes, stress response genes, or in other PD-related genes identified by genome-wide association studies (GWAS) in authentic midbrain dopaminergic cultures. This is in contrast to our ability to identify a phenotype in *PARK2* mutant lines at the same stage [31]. Interestingly, levels of *SNCA* transcript were different in the two isogenic lines. One line showed a three-fold higher transcript level (A6), while the other had levels indistinguishable from the control. However, even in this case, we could not glean any additional insight into the behavior of the lines or the signaling pathways that may underlie the disease. Our results suggest that SNCA mutations increase the probability of the disease, but require cofactors that our current analysis is not able to detect. Overall, our data suggests that *in vitro* culture variability and the inherent variability of biological systems may drown out causative signals of disease process in some monogenetic disorders. This raises doubts that causality can be determined using unbiased functional screening alone in such monogenic lines.

In an attempt to further refine the system, we reasoned that stressing it might reveal differences between *SNCA* triplication and control dopaminergic cultures. We used an MPTP-based model that we have previously successfully utilized for a screen [39] to analyze gene expression in dopaminergic cultures derived from the two SNCA clones. Both lines showed similar alterations to the controls in mitochondrial genes, stress response, and complex 1 (Table 5), forbidding the discovery of a novel pathway uniquely activated in *SNCA* triplication line. Thus, despite our efforts to minimize variability due to line generation and cell culture, and after adding the external stress to the system, we were unable to pinpoint a unique difference between the normal and mutant lines.

Another group has generated iPSC lines carrying the *SNCA* triplication [14]. Despite difference in methods of iPSC generation and dopaminergic differentiation, overall results were largely similar. Like this report, authors observed variability among clones, but all clones retained the ability to differentiate into neurons, including dopaminergic neurons. No significant changes in undifferentiated cells were seen, though SNCA aggregation was seen in a small number of TH-negative cells after prolonged culture. Consistent with our results, increased cell death was observed only in response to external stress, and was not detected under normal conditions. Our results extend these observations by examining a larger panel of genes and confirming the response of *SNCA* mutants to MPTP and in particular the potential importance of Harakari in the death response.

It has been suggested that *SNCA* overexpression or mutations may alter the innate immune response and that this may be related to epigenetic modulation of the inflammasome response [47–49]. We note that the most significant changes in gene expression data from all PD-derived dopamine neurons were changes in the interferon transcript and in the neuronal inflammasome *NLRP2*. These changes have been implicated in the progression of the disease [50] and will be the focus of future analysis.

We utilized two strategies in an attempt to determine if iPSC lines we generated could be useful for the discovery of novel pathways that contribute to PD. One was to generate an isogenic control and use that as a sensitive indicator of likely changes that could be independently confirmed in the patient lines and by other independent tests. This was successful with the *PARK2* mutant lines and has been reported elsewhere [31,51]. As the second strategy, we attempted to extract signal from noise by using a biased search strategy based on published literature on mechanism of action of the protein and the known association of genes with PD from GWAS. We reasoned that all monogenic disorders lead to a loss of PD and this loss appears similar to that seen after mitochondrial damage. We examined the expression of genes known to be associated with PD at the dopaminergic neuron stage, but rather than an unbiased analysis of differentially expressed genes, we focused on genes that are known to be associated with PD. This indicated that *PARK2* and *PINK1* interact with HTRA2/OMNI and ATP132, and that the lysosomal pathway and lipid metabolism genes may be important in mitophagy. Changes in *LRRK2* and *SNCA* in lines carrying *GBA* and *PARK2* mutants were subtle and variable, suggesting these are independent or parallel pathways (S6 Table). These inferences provide important clues as what to assess in subsequent experiments.

## Conclusions

In summary, our studies suggest that using single iPSC lines for drug screens in a monogenic disorder with a well-characterized phenotype may not be sufficient to determine causality and mechanism of action due to the inherent variability of biological systems. Developing a database to increase the number of lines, stressing the system, using isogenic controls, and using more focused strategies for analyzing large scale data sets would reduce the impact of line-to-line variations and may provide important clues to the etiology of PD. In an attempt to enable such a large-scale analysis, we will deposit the lines in a suitable repository for widespread use and the datasets will be made widely available via the NCBI database.

## Supporting Information

S1 Fig Characterization of each derived PD patient iPSC and NSC line. A: Immunocytochemistry for pluripotency factors OCT4, NANOG, SOX2, and TRA1-60. B: Alkaline phosphatase reactivity. Inserts: Images taken at higher magnification. C: Immunocytochemistry for markers of the three germ layers in embryoid bodies. Scale bar as marked. D: Karyotype analysis. E: STR profiles of parent fibroblast and iPSC. F: Immunocytochemistry NSCs with antibodies against NSC markers SOX1, NESTIN, and PAX6. G: Immunocytochemistry for dopaminergic (TH and LMX1A), and midbrain (FOXA2) markers. Scale bar as marked. S1A characterization of line A23, S1B characterization of line K20, S1C characterization of line K25, S1D characterization of line T101, S1E characterization of line I3, S1F characterization of line Y9, S1G characterization of line S110 and S1H characterization of line B119.
(TIF)

S2 Fig Dopaminergic differentiation of α-synuclein (SNCA) triplication lines relative to control line. A: Immunocytochemistry for TH/β-III-tubulin and TH/SNCA in control and SNCA triplication line demonstrates SNCA expression in TH-positive neurons (arrow heads), as well as some TH-negative cells (asterisk). B: qPCR analysis of *SNCA* gene expression in control (L1 and Y9) and SNCA triplication lines. *TBP* was used as a reference gene, and data are normalized to Y9 *SNCA* expression level. C: Western blot validation of elevated expression of alpha-synuclein in two SNCA cell lines (A6 and A23) relative to two control lines (L1 and Y9). TH and LMX1A were used to confirm dopaminergic differentiation in cultures. β-actin (ACTB) was used as a loading control.
(TIF)

S1 Table List of primers used in the study. S1H Fig. Characterization of patient-specific PD line, B119. A: Immunocytochemistry for pluripotency factors OCT4, NANOG, SOX2, and TRA1-60. B: Alkaline phosphatase reactivity. Inserts: Images taken at higher magnification. C: Immunocytochemistry for markers of the three germ layers in embryoid bodies. Scale bar as marked. D: Karyotype analysis. E: STR profiles of parent fibroblast and iPSC. F: Immunocytochemistry NSCs with antibodies against NSC markers SOX1, NESTIN, and PAX6. G: Immunocytochemistry for dopaminergic (TH and LMX1A), and midbrain (FOXA2) markers. Scale bar as marked.
(DOCX)

S2 Table Short tandem repeat (STR) profiles of Parkinson’s disease patient fibroblasts and corresponding iPSC lines. (DOCX)

S3 Table The list of differentiated iPSC clones into DA neurons that were analyzed by microarrays. (DOCX)

S4 Table The coefficient of correlation values within distinct developmental groups. Excel file.
(XLSX)

S5 Table QC list for all stages. Excel file.
(XLSX)

S6 Table Gene expression of PD related genes from PD dopamine cultures. (DOCX)
