# Supplementary figures and images for: Derivation, Characterization, and Neural Differentiation of Integration-Free Induced Pluripotent Stem Cell Lines from Parkinson’s Disease Patients Carrying SNCA, LRRK2, PARK2, and GBA Mutations
Source: PLoS One. 2016 May 18;11(5):e0154890. doi: 10.1371/journal.pone.0154890 (PMC4871453; doi:10.1371/journal.pone.0154890)

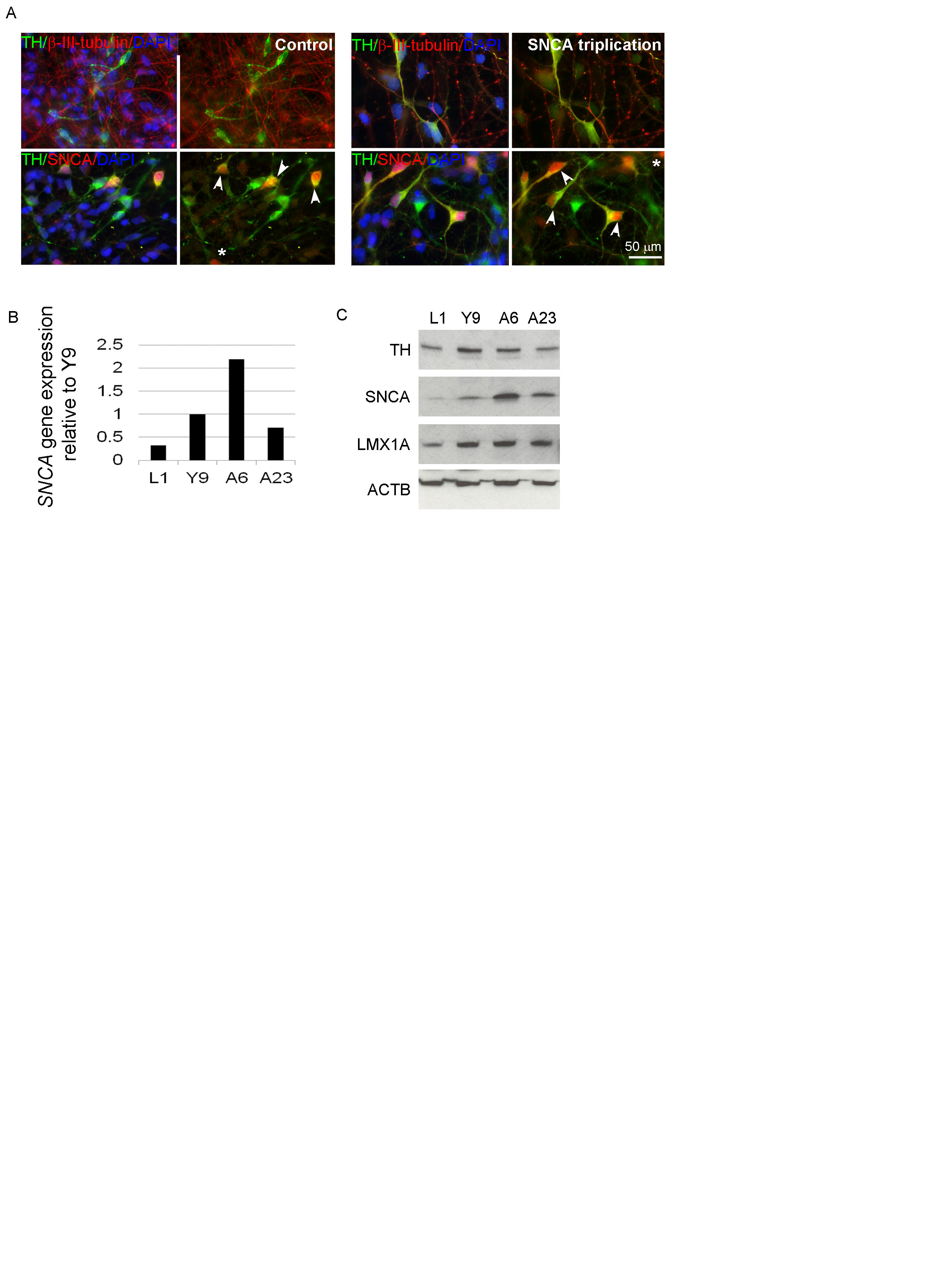

Supplement: S2 Fig — A: Immunocytochemistry for TH/β-III-tubulin and TH/SNCA in control and SNCA triplication line demonstrates SNCA expression in TH-positive neurons (arrow heads), as well as some TH-negative cells (asterisk). B: qPCR analysis of SNCA gene expression in control (L1 and Y9) and SNCA triplication lines. TBP was used as a reference gene, and data are normalized to Y9 SNCA expression level. C: Western blot validation of elevated expression of alpha-synuclein in two SNCA cell lines (A6 and A23) relative to two control lines (L1 and Y9). TH and LMX1A were used to confirm dopaminergic differentiation in cultures. β-actin (ACTB) was used as a loading control. (TIF) [file pone.0154890.s002.tif]
