# Supplementary material for: Derivation, Characterization, and Neural Differentiation of Integration-Free Induced Pluripotent Stem Cell Lines from Parkinson’s Disease Patients Carrying SNCA, LRRK2, PARK2, and GBA Mutations
Source: PLoS One. 2016 May 18;11(5):e0154890. doi: 10.1371/journal.pone.0154890 (PMC4871453; doi:10.1371/journal.pone.0154890)
Supplement: S1 Table — A: Immunocytochemistry for pluripotency factors OCT4, NANOG, SOX2, and TRA1-60. B: Alkaline phosphatase reactivity. Inserts: Images taken at higher magnification. C: Immunocytochemistry for markers of the three germ layers in embryoid bodies. Scale bar as marked. D: Karyotype analysis. E: STR profiles of parent fibroblast and iPSC. F: Immunocytochemistry NSCs with antibodies against NSC markers SOX1, NESTIN, and PAX6. G: Immunocytochemistry for dopaminergic (TH and LMX1A), and midbrain (FOXA2) markers. Scale bar as marked. (DOCX) [file pone.0154890.s003.docx]

**S1 Table. List of primers used in the study.**

| Target | Forward primer | Reverse primer |
| --- | --- | --- |
| oct 4 endo | Cctcacttcactgcactgta | caggttttctttccctagct |
| oct4 total | Agcgaaccagtatcgagaac | ttacagaaccacactcggac |
| sox 2 endo | Cccagcagacttcacatgt | cctcccatttccctcgtttt |
| sox2 total | Agctacagcatgatgcagga | ggtcatggagttgtactgca |
| Nanog | tgaacctcagctacaaacag | tggtggtaggaagagtaaag |
| sev | GGATCACTAGGTGATATCGAGC | ACCAGACAAGAGTTTAAGAGATATGTATC |
| DAT | CCTGCTCTTCATGGTCATTG | AAGCCCACACCTTTCAGTAT |
| VMAT2 | TGGATTCGTCAATGATGCCT | CAGAAGGACCTATAGCATACCC |
| DCX | AATCCCAACTGGTCTGTCAAC | gtttcccttcatgactcggca |
| TH | GTGCTAAACCTGCTCTTCTC | GCTTCAAACGTCTCAAACAC |
| nnat | ATCATCGGCTGGTACATCTTCC | GACACCGTGTATGCCAGCTTCT |
| DPP7 | TCAGTGCCTACCTGAGGATGAAGT | AAGTCCTTGATCTGTCGGAACGCT |
| snca | ACCAAACAGGGTGTGGCAGAAG | CTTGCTCTTTGGTCTTCTCAGCC |
| GAPDH | gatgacatcaagaaggtggtga | gtctacatggcaactgtgagga |
| ACTB | TGAAGTGTACGTGGACATC | GGAGGAGCAATGATCTTGAT |

- *TBP, EN2, LMX1B, GIRK2, NURR1, TUBB3, GFAP, OLIG2, SOX1, PAX6, SLC6A4*, gDNA contamination, RQ1 and RQ2 (RNA quality) primer assays were purchased from Bio-Rad.
