# Supplementary material for: Derivation, Characterization, and Neural Differentiation of Integration-Free Induced Pluripotent Stem Cell Lines from Parkinson’s Disease Patients Carrying SNCA, LRRK2, PARK2, and GBA Mutations
Source: PLoS One. 2016 May 18;11(5):e0154890. doi: 10.1371/journal.pone.0154890 (PMC4871453; doi:10.1371/journal.pone.0154890)
Supplement: S2 Table — (DOCX) [file pone.0154890.s004.docx]

S2 Table. Short tandem repeat (STR) profiles of Parkinson’s disease patient fibroblasts and corresponding iPSC lines.

|  | Control | | SNCA | | PARK2 | | | | | | | | LRRK2 | | | GBA | |
| --- | --- | --- | --- | --- | --- | --- | --- | --- | --- | --- | --- | --- | --- | --- | --- | --- | --- |
| Locus | **Y**  **Fibro** | **Y9**  **iPSC** | **A**  **fibro** | **A6**  **iPSC** | **P**  **fibro** | **P1**  **iPSC** | **I**  **Fribro** | **I3**  **iPSC** | **S**  **fibro** | **S110**  **iPSC** | **B**  **fibro** | **B119**  **iPSC** | **K**  **fibro** | **K20**  **iPSC** | **K25**  **iPSC** | **T**  **fibro** | **T101**  **iPSC** |
| AMEL | X | X | X | X | X, Y | X, Y | X, Y | X, Y | X | X | X | X | X, Y | X, Y | X, Y | X, Y | X, Y |
| CSF1PO | 10, 11 | 10, 11 | 12 | 12 | 10, 11 | 10, 11 | 10, 11 | 10, 11 | 12,13 | 12,13 | 10 | 10 | 10, 12 | 10, 12 | 10, 12 | 10, 14 | 10, 14 |
| D13S317 | 12 | 12 | 9, 11 | 9, 11 | 11, 12 | 11, 12 | 9, 12 | 9, 12 | 11,12 | 11,12 | 11,12 | 11,12 | 9, 12 | 9, 12 | 9, 12 | 9, 11 | 9, 11 |
| D16S539 | 11 | 11 | 11, 12 | 11, 12 | 10, 11 | 10, 11 | 11, 12 | 11, 12 | 11 | 11 | 12,13 | 12,13 | 8, 13 | 8, 13 | 8, 13 | 12 | 12 |
| D21S11 | 28 | 28 | 30, 31.2 | 30, 31.2 | 28, 32.2 | 28, 32.2 | 29 | 29 | 29 | 29 | 29,30 | 29,30 | 29, 30 | 29, 30 | 29, 30 | 28, 31 | 28, 31 |
| D5S818 | 11, 13 | 11, 13 | 11 | 11 | 11, 13 | 11, 13 | 11, 12 | 11, 12 | 10 | 10 | 11 | 11 | 11, 12 | 11, 12 | 11, 12 | 11, 12 | 11, 12 |
| D7S820 | 9, 10 | 9, 10 | 9, 11 | 9, 11 | 9, 11 | 9, 11 | 8, 10 | 8, 10 | 8,10 | 8,10 | 10,11 | 10,11 | 10, 11 | 10, 11 | 10, 11 | 10, 11 | 10, 11 |
| TH01 | 9, 9.3 | 9, 9.3 | 7, 9.3 | 7, 9.3 | 8, 9.3 | 8, 9.3 | 6, 9.3 | 6, 9.3 | 7,9 | 7,9 | 8,9.3 | 8,9.3 | 7, 9 | 7, 9 | 7, 9 | 7, 8 | 7, 8 |
| TPOX | 8, 11 | 8, 11 | 8, 11 | 8, 11 | 8 | 8 | 8, 10 | 8, 10 | 8,9 | 8,9 | 8,12 | 8,12 | 8, 9 | 8, 9 | 8, 9 | 8 | 8 |
| vWA | 16, 17 | 16, 17 | 15, 18 | 15, 18 | 16, 17 | 16, 17 | 14, 17 | 14, 17 | 16 | 16 | 16 | 16 | 17, 18 | 17, 18 | 17, 18 | 16, 17 | 16, 17 |
