# Supplementary material for: Derivation, Characterization, and Neural Differentiation of Integration-Free Induced Pluripotent Stem Cell Lines from Parkinson’s Disease Patients Carrying SNCA, LRRK2, PARK2, and GBA Mutations
Source: PLoS One. 2016 May 18;11(5):e0154890. doi: 10.1371/journal.pone.0154890 (PMC4871453; doi:10.1371/journal.pone.0154890)
Supplement: S3 Table — (DOCX) [file pone.0154890.s005.docx]

S3 Table. The list of differentiated iPSC clones into DA neurons that were analyzed by microarrays.

|  | **Parent fibroblast** | **Clones** | **Mutation** |
| --- | --- | --- | --- |
| **SNCA** | **A** | A6, A23 | SNCA triplication |
| **PARK2** | **P** | P1 | PARK2: R42P PARK2: EX3DEL |
|  | **I** | I3 | PARK2: EX3-4DEL PARK2: 1-BP DEL, 255A |
|  | **B** | B119 | PARK2: R275W |
|  | **S** | S110 | PARK2: R42P |
| **LRRK2** | **K** | K20, K25 | LRRK2: G2019S |
| **GBA** | **T** | T101 | GBA: N370S |
| **Control** | **Y** | Y9 | Population-control |
