# Supplementary material for: Derivation, Characterization, and Neural Differentiation of Integration-Free Induced Pluripotent Stem Cell Lines from Parkinson’s Disease Patients Carrying SNCA, LRRK2, PARK2, and GBA Mutations
Source: PLoS One. 2016 May 18;11(5):e0154890. doi: 10.1371/journal.pone.0154890 (PMC4871453; doi:10.1371/journal.pone.0154890)
Supplement: S6 Table — (DOCX) [file pone.0154890.s008.docx]

**S6 Table. Gene expression of PD related genes from PD dopamine cultures.**

| SYMBOL | Y9 DA | P1 DA | I3 DA | S110 DA | B119 DA | A6 DA | A23 DA | K20 DA | K25 DA | T101DA2 |
| --- | --- | --- | --- | --- | --- | --- | --- | --- | --- | --- |
| ACMSD | -8.21 | -9.58 | -3.87 | -12.79 | -14.81 | -1.00 | -16.51 | -11.66 | -1.79 | -8.58 |
| ADH1C | 20.37 | -7.45 | 2.21 | -0.10 | -11.25 | -7.61 | -4.61 | -8.84 | 1.66 | -19.04 |
| ATP13A2 | 485.53 | 260.21 | 302.00 | 619.47 | 599.92 | 370.56 | 793.64 | 321.72 | 385.49 | 463.15 |
| ATXN2 | 4445.01 | 3519.47 | 3811.41 | 4891.84 | 4671.98 | 3492.05 | 4196.41 | 3466.75 | 4555.72 | 6047.03 |
| ATXN8OS | -20.37 | -24.56 | -16.67 | -17.03 | -26.46 | -24.98 | -10.75 | -27.66 | -20.78 | -25.26 |
| BST1 | 6.41 | 8.98 | -11.68 | 2.81 | 4.81 | 7.59 | 0.64 | 29.12 | 20.40 | -5.39 |
| EIF4G1 | 655.12 | 606.44 | 663.95 | 560.28 | 706.38 | 631.62 | 1022.91 | 605.62 | 586.24 | 670.74 |
| FBXO7 | 407.06 | 407.73 | 414.82 | 497.01 | 360.94 | 357.82 | 476.83 | 504.59 | 447.10 | 494.93 |
| FGF20 | 14.06 | 17.60 | 23.09 | 21.65 | 41.71 | 31.48 | 18.39 | 10.31 | 19.73 | 24.81 |
| GAK | 451.93 | 521.67 | 518.23 | 587.91 | 605.91 | 442.56 | 493.92 | 526.03 | 496.18 | 482.07 |
| GBA | 474.64 | 420.98 | 647.33 | 535.61 | 537.77 | 469.29 | 667.38 | 396.07 | 445.45 | 342.05 |
| GIGYF2 | 11.73 | 31.18 | 15.23 | 16.25 | 11.01 | 20.61 | 15.40 | 32.72 | 18.77 | 11.39 |
| GPNMB | 30.77 | -0.54 | -0.87 | 14.42 | 15.25 | 0.51 | -7.42 | -4.81 | -4.38 | 10.99 |
| HIP1R | 54.85 | 45.51 | 47.73 | 74.15 | 64.46 | 77.75 | 93.39 | 20.09 | 69.20 | 143.43 |
| HTRA2 | 531.29 | 551.39 | 482.23 | 581.89 | 651.42 | 605.71 | 747.96 | 524.61 | 552.96 | 609.14 |
| LRRK2 | 15.13 | -0.82 | 12.67 | 5.50 | 9.48 | 4.04 | 12.95 | 3.78 | 14.48 | -0.22 |
| MAPT | 2899.65 | 1461.09 | 1802.55 | 3453.97 | 7128.35 | 2688.02 | 2727.37 | 1098.19 | 2050.17 | 4376.43 |
| MC1R | 315.76 | 267.26 | 502.72 | 515.94 | 423.32 | 393.80 | 303.73 | 194.79 | 316.56 | 303.76 |
| MCCC1 | 911.07 | 1065.81 | 929.28 | 722.33 | 1048.20 | 978.78 | 957.72 | 757.02 | 960.53 | 846.63 |
| MED13 | 19.21 | 29.25 | 22.97 | 23.20 | 45.60 | 35.35 | 20.86 | 29.56 | 43.62 | 35.37 |
| PARK2 | 25.96 | 14.50 | 8.98 | 22.60 | 12.58 | 20.26 | 19.86 | 12.92 | 6.80 | 8.81 |
| PARK7 | 18646.44 | 17980.58 | 20628.00 | 17880.19 | 19446.36 | 18618.01 | 18861.67 | 16723.00 | 16835.48 | 14731.54 |
| PDXK | 2311.02 | 1571.32 | 2264.98 | 2432.50 | 3756.65 | 1810.69 | 2487.26 | 1131.60 | 1989.89 | 2051.46 |
| PINK1 | 1657.08 | 942.26 | 1326.78 | 1475.03 | 1517.90 | 1240.76 | 1461.49 | 960.99 | 1064.91 | 1363.52 |
| PLA2G6 | 11.89 | 27.80 | 26.60 | 32.77 | 17.87 | 22.09 | 46.12 | 23.42 | 22.51 | 41.95 |
| PM20D1 | 23.58 | 37.58 | 46.96 | 66.92 | 72.62 | 24.47 | 34.32 | 43.06 | 62.21 | 39.48 |
| RAB25 | 15.62 | 17.81 | 29.09 | 21.00 | 26.32 | 14.94 | 11.15 | 12.63 | 22.66 | 30.00 |
| SETD1A | 403.10 | 367.85 | 288.51 | 388.43 | 380.22 | 454.33 | 807.26 | 479.79 | 404.92 | 511.10 |
| SNCA | 2294.03 | 790.67 | 934.73 | 3375.57 | 7063.93 | 4945.51 | 2470.21 | 1700.34 | 1150.25 | 1898.23 |
| STK39 | 4170.62 | 4019.82 | 2332.23 | 4620.34 | 4959.62 | 4530.63 | 3252.53 | 4296.89 | 3880.28 | 4493.47 |
| TBP | 1093.25 | 937.57 | 996.00 | 1041.39 | 1194.09 | 1095.82 | 944.08 | 1013.34 | 1016.98 | 958.00 |
| UCHL1 | 30863.32 | 16134.22 | 20129.27 | 33073.12 | 35416.15 | 25723.03 | 24286.75 | 17646.29 | 20242.46 | 24408.47 |
| VPS35 | 5890.16 | 5640.57 | 5909.77 | 6363.11 | 6152.16 | 5949.14 | 4108.47 | 5510.44 | 5043.35 | 4464.64 |
